# A Cyclodextrin‐Stabilized Spermine‐Tagged Drug Triplex that Targets Theophylline to the Lungs Selectively in Respiratory Emergency

**DOI:** 10.1002/adtp.202000153

**Published:** 2020-09-25

**Authors:** Zarif M. Sofian, Faiza Benaouda, Julie Tzu‐Wen Wang, Yuan Lu, David J. Barlow, Paul G. Royall, Doaa B. Farag, Khondaker Miraz Rahman, Khuloud T. Al‐Jamal, Ben Forbes, Stuart A. Jones

**Affiliations:** ^1^ School of Cancer and Pharmaceutical Sciences Faculty of Life Sciences & Medicine King's College London Franklin‐Wilkins Building, 150 Stamford Street London SE1 9NH UK; ^2^ Department of Pharmaceutical Technology Faculty of Pharmacy Universiti Malaya Kuala Lumpur 50603 Malaysia; ^3^ Faculty of Pharmacy Misr International University Cairo 11431 Egypt

**Keywords:** cyclodextrin, emergency medicine, ion‐pairing, lung targeting, triplexes

## Abstract

Ion‐pairing a lifesaving drug such as theophylline with a targeting moiety could have a significant impact on medical emergencies such as status asthmaticus or COVID‐19 induced pneumomediastinum. However, to achieve rapid drug targeting in vivo the ion‐pair must be protected against breakdown before the entry into the target tissue. This study aims to investigate if inserting theophylline, when ion‐paired to the polyamine transporter substrate spermine, into a cyclodextrin (CD), to form a triplex, could direct the bronchodilator to the lungs selectively after intravenous administration. NMR demonstrates that upon the formation of the triplex spermine protruded from the CD cavity and this results in energy‐dependent uptake in A549 cells (1.8‐fold enhancement), which persists for more than 20 min. In vivo, the triplex produces a 2.4‐fold and 2.2‐fold increase in theophylline in the lungs 20 min after injection in rats and mice, respectively (*p* < 0.05). The lung targeting is selective with no increase in uptake into the brain or the heart where the side‐effects of theophylline are treatment‐limiting. Selectively doubling the concentration of theophylline in the lungs could improve the benefit‐risk ratio of this narrow therapeutic index medicine, which continues to be important in critical care.

## Graphical abstract

This article demonstrates that ion‐pairing theophylline, a drug used in medical emergencies such as status asthmaticus or COVID‐19 induced pneumomediastinum, with a polyamine, can direct delivery to the lung. Such an approach is effective when the ion‐pair is stabilized as a triplex with cyclodextrin, which increases the in vivo ion‐pair life‐time to facilitate active accumulation in the lung.

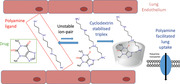

## 1 Introduction

Linking drugs to targeting ligands can achieve preferential accumulation of therapeutic agents in target tissues after administration.^[^ ^1^ ^]^ This approach can reduce the impact of “off‐target” drug side‐effects and help retain the drug at its site of action.^[^ ^1^ ^]^ However, it remains problematic to link targeting ligands to many low molecular weight actives in a manner that does not compromise their pharmacological activity.^[^ ^2^ ^]^ For example, polyamine targeting ligands are highly effective in targeting small molecular weight anticancer drugs to tumors, but their covalent linkage to the targeted drugs modifies their overall properties such that their anticancer activity is diminished.^[^ ^3^ ^]^ Likewise, the loading of small molecular weight drugs into targeting ligand decorated carriers can protect the drug and improve its cellular uptake, but the process of carrier release can limit the rapidity of drug action.^[^ ^4^ ^]^ This renders many targeting strategies unsuitable for the treatment of acute conditions, i.e., medical emergencies, which is a field that lacks bespoke drug delivery systems.^[^ ^5^ ^]^

Published work has demonstrated that ion‐pairing theophylline, a drug that is commonly used in medical emergencies such as status asthmaticus or COVID‐19 related pneumomediastinum, to the polyamine transporter substrate (PTS), with spermine, enhanced its lung uptake by 3.6‐fold in a rat isolated perfused lung model.^[^ ^6^ ^]^ Attaching the targeting ligand to the drug through ion‐pair formation has the advantage that the dissociation rate of an ion‐paired system is controlled by the complex association strength. Therefore, the use of ion‐pairs with moderate association strength avoids any deleterious effect on drug pharmacological activity.^[^ ^7^ ^]^ However, the effects obtained using an ion‐paired polyamine targeting ligand were short‐lived due to the rapid dissociation kinetics of the theophylline‐spermine ion‐pair which indicated that more work is needed to understand ion‐pair half‐life in vivo.

Experimentally it is very difficult to directly measure ion‐pair half‐life in biological fluids using traditional spectroscopic methods of analysis. However, in vitro and ex vivo functional studies suggest that a drug linked to a targeting ligand through the formation of an ion‐pair can remain associated for more than 30 min in biological fluids.^[^ ^8^ ^]^ This is significantly longer than simple electrolyte ion‐pairs which break down in ≈1 ns.^[^ ^9^ ^]^ Also, the physical stability of ion‐pairs could be prolonged through association with stabilizing biomolecules such as cyclodextrins (CD). CDs are cyclic (α‐1,4)‐linked‐oligosaccharides that display a cone‐shape with a hollow tapered cavity. The three most common types of CDs are α‐, β‐, and γ‐CDs. They comprise 6, 7, and 8 glucopyranose units, respectively. The exterior surface is hydrophilic while the central cavity is relatively hydrophobic.^[^ ^10^, ^11^ ^]^ Through a host‐guest complexation, both native CD and their derivatives can encapsulate lipophilic molecules or lipophilic parts of the molecules.^[^ ^12^ ^]^ If CDs could form inclusion complexes with ion‐pairs to form a triplex they could stabilize a drug‐targeting ion‐pair and thus be used to tune the ability of the targeting ligand to deliver the drug by modifying the dynamics of dissociation in vivo.^[^ ^13^ ^]^

This study aimed to determine whether supramolecular triplex assemblies of drug‐targeting ligand and CD could be used to direct drug delivery in a manner that would apply to the treatment of serious acute medical conditions. Theophylline, a drug used to open the airways in critical care, commercially formulated at pH 9.6 for intravenous administration e.g., Phyllocontin, was used because directing this agent toward the lungs could be clinically beneficial.^[^ ^14^, ^15^, ^16^ ^]^ Additionally, previous work has shown that theophylline can ion‐pair with the polyamine transport system (PTS) substrate spermine.^[^ ^6^ ^]^ In silico studies were conducted to understand the molecular structure of the triplex, in vitro studies were used to demonstrate theophylline ion‐pair system uptake into A549 cells when formulated as the CD ion‐pair complex and in vivo studies showed how this influenced drug biodistribution.

## 2 Results

### 2.1 Theophylline‐CD Complex Characterization

**Figure 1:**
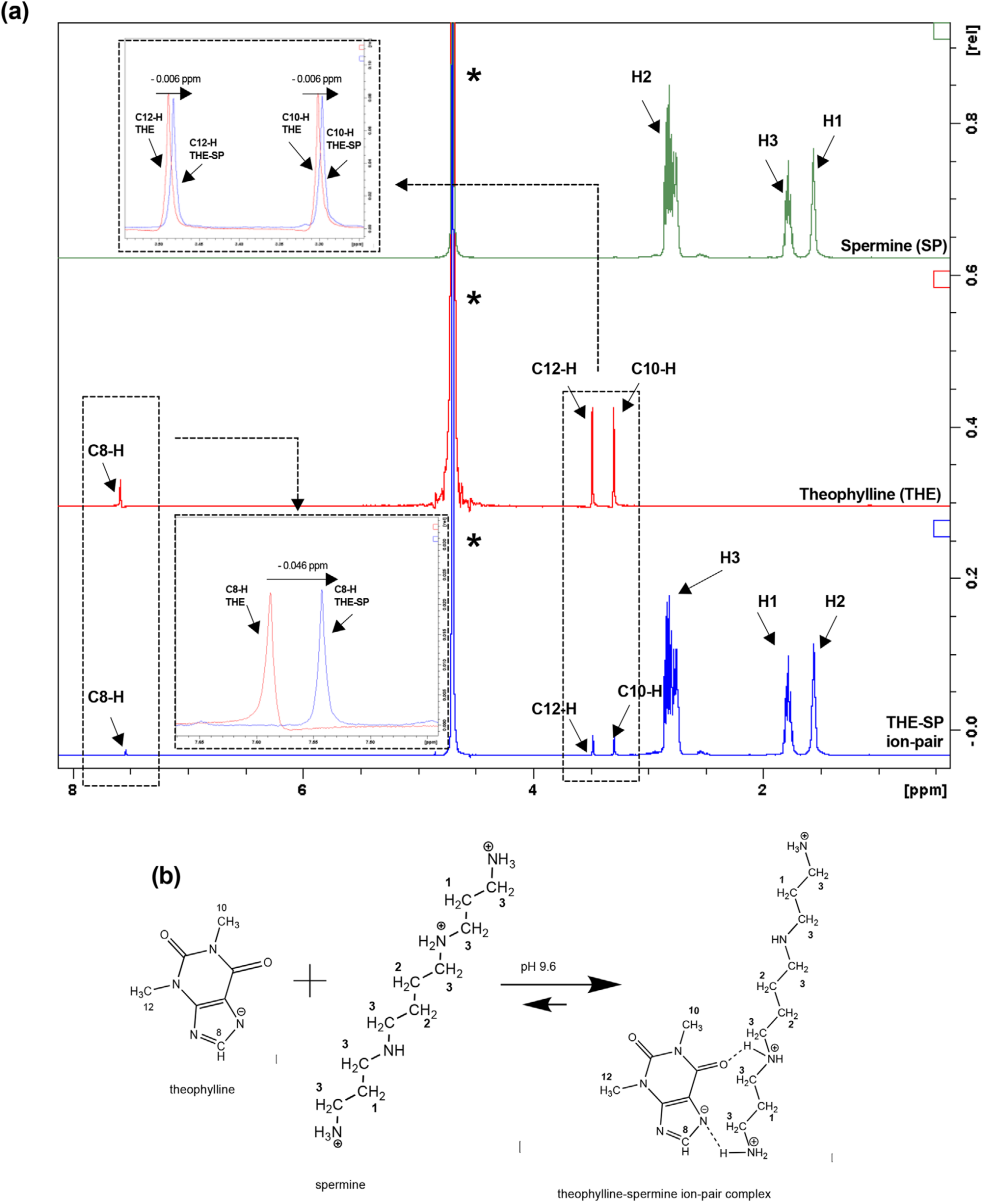
a) The ^1^H‐NMR spectra of 0.1 m spermine (top graph), 0.005 m theophylline (middle graph) and theophylline‐spermine 0.005:0.1 m mixture (bottom graph) in D_2_O pH 9.6 ± 0.1 b) the proposed molecular structure of the ion‐pair complex. Dotted squares indicate regions of possible hydrogen bonding formation. *asterisk highlights the D_2_O signal.

The NMR characterization of theophylline and spermine showed spontaneous formation ion‐pairs in the presence of excess counterion. The NMR upfield shift of the theophylline peaks C_8_‐H (7.589 ppm), C_10_‐H (3.303 ppm), and C_12_‐H (3.489 ppm), when mixed with spermine (**Figure** **1**), suggested hydrogen bond formation between the N_7_ and carbonyl groups of theophylline with the secondary and primary amines of spermine respectively in the ion‐pairs. The interactions with spermine occurred at the terminal positions due to the variations in the pKa's along the molecule, which made interactions in these positions more favorable. The NMR data were in agreement with previous infrared spectroscopy studies, which also suggested the formation of 2 hydrogen bonds between theophylline and spermine.^[^ ^6^ ^]^

The largest peak shift in the NMR spectra was observed for the theophylline C_8_‐H peak and this was used to construct the theophylline‐spermine association curve and derive the conditional association constant for the theophylline‐spermine ion‐pair, pK_NMR_ = 1.5 ± 0.02 (see Figure S1, Supporting Information). This conditional association constant was higher than the association constant generated by infrared spectroscopy, pK_FTIR_ at 0.9 ± 0.1, reported in previous work.^[^ ^6^ ^]^ Of the two values the pK_NMR_ was considered to be the most accurate because although the infrared studies directly measured the N_7_ involved in the hydrogen bonding to spermine, the theophylline tautomerism complicated the infrared spectra. The same problems were not experienced with the NMR data.^[^ ^17^ ^]^ The affinity constants of the ion‐pairs were consistent with previously reported estimates for ion‐pairs formed with two hydrogen bonds (affinity constants of 0.8^[^ ^6^ ^]^ and 1.1^[^ ^7^ ^]^).

**Figure 2:**
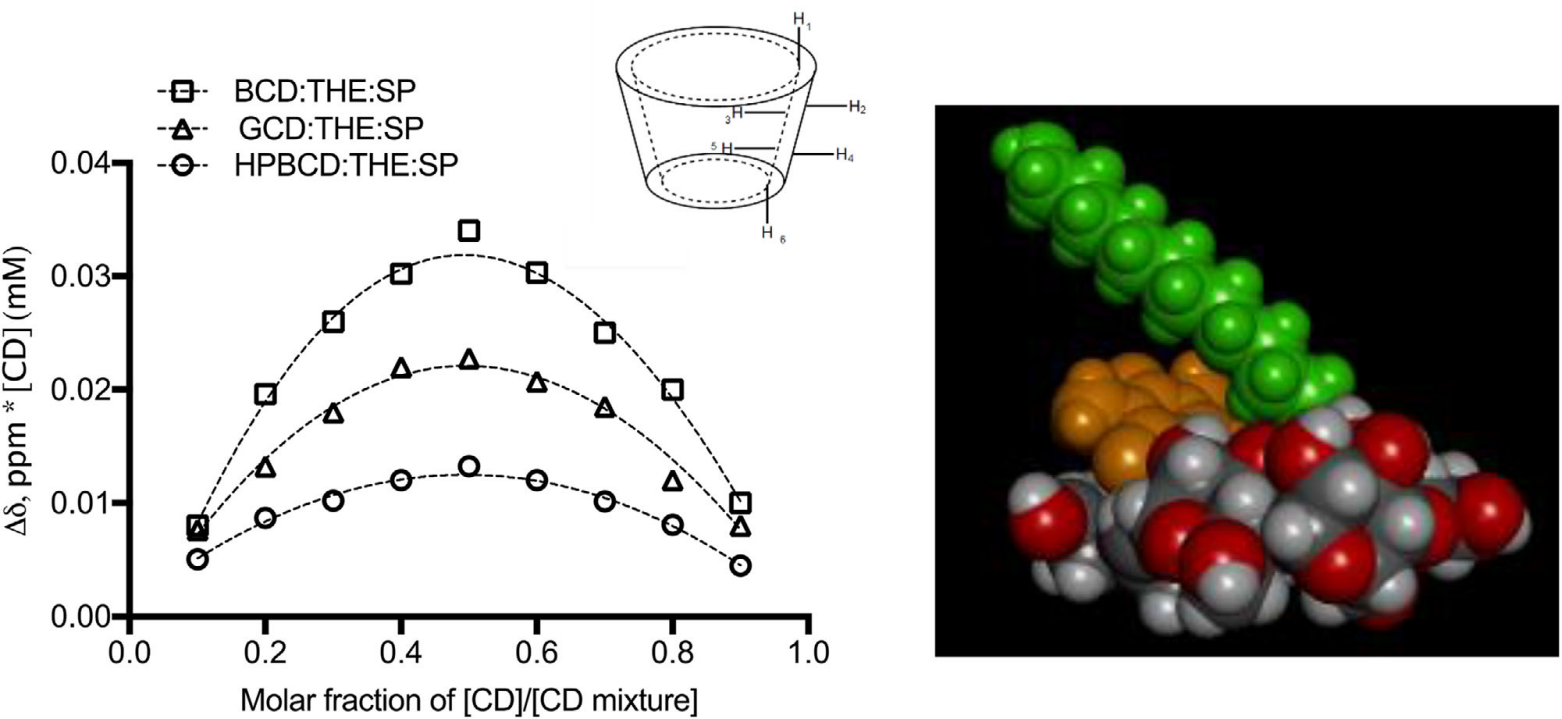
Left: Job's plots of β‐cyclodextrin (BCD), γ‐cyclodextrin (GCD) and HP‐β‐cyclodextrin (HPBCD) complexed with theophylline‐spermine (1:20 molar ratio) at pH 9.6 ± 0.1 using the chemical shift of H3 cyclodextrins. Centre: cyclodextrin cavity showing the proton locations (taken from ^[^ ^18^ ^]^). Right: The structure of a 1:1 βCD‐[THE‐SP] complex in water at pH 9.6, blue = nitrogen, red = oxygen, white = hydrogen, gray = carbon, green was spermine, orange was theophylline, and cyclodextrin was indicated by red and gray. Images were generated using Discovery Studio Visualizer (Accelrys Inc, CA, USA).

The addition of CD to the theophylline‐spermine ion‐pairs resulted in upfield shifts in all the NMR hydrogens signals of the CD, i.e., β‐CD, γ‐CD, and HP‐β‐CD (see Table S1, Supporting Information). Incrementally greater shifts were observed in response to increasing concentration of the complex and the shifts were greater in magnitude than the digital resolution of the NMR, which was 0.0006 ppm. In addition, the shifts were of a similar magnitude to those recorded for the spermine‐theophylline ion‐pair formation, thus it was concluded that the NMR peak shifts demonstrated that CD‐ion‐pair host–guest complex had formed. The shifts for β‐CD (e.g., H3 – 0.052 ppm) were of the greatest magnitude when mixed with the theophylline ion‐pair followed by γ‐CD (e.g., H3 – 0.044 ppm) and then HP‐β‐CD (e.g., H3 – 0.022 ppm) suggesting the rank order of intermolecular interaction strength was β‐CD > γ‐CD > HP‐β‐CD. For β‐CD the proton shifts were greater for the hydrogens that were located in the inner cavity, i.e., H‐3 (−0.052 ppm) and H‐5 (−0.046 ppm), compared to those located more distally, e.g., H‐6 (−0.03 ppm), H‐1 (0.036 ppm), H‐2 (−0.035 ppm), and H‐4 (−0.038 ppm). A similar trend in terms of the hydrogens in the inner cavity was also observed for the ion‐pair complexes with γ‐CD and HP‐β‐CD (**Figure** **2**).

Several of the proton signals for both of theophylline and spermine showed larger peak shifts compared to NMR of the ion‐pair when mixed with CD, e.g., the theophylline C_12_ showed a shift of – 0.030 ppm and at C_10_ – 0.028 ppm, which supported the notion both of the molecules in the ion‐pair penetrated the β‐CD cavity. No peak broadening was observed in the theophylline and spermine NMR signals in the presence of the CD, which suggested that any changes to the sample viscosity or the increase in the complex molecular weight did not modify the peaks, an effect that aligned with previous published NMR results assessing CD‐drug interactions.^[^ ^19^ ^]^ The Job's plot constructed using the H‐3 signal of the CDs demonstrated that the stoichiometry between the ion pair and the CD was 1:1, i.e., the plot max was at *r* = 0.5 (Figure 2 and Table S2, Supporting Information).

Using molecular modeling it was evident that only ≈10% of the spermine total volume and ≈10% of the theophylline volume could be inserted into the β‐CD cavity (Figure 2). The theophylline and spermine could not penetrate the β‐CD cavity to any greater depth, because the sum of their cross‐sectional areas at several points in the complex (individually ≈28 and 25 Å^2^, respectively) exceeded the cross‐sectional area of the cavity (42 Å^2^). In this (energy minimized) model the theophylline‐spermine N_7_….H distance was 2.2 Å. Although CD has previously been employed to stabilize metal complexes,^[^ ^20^, ^21^ ^]^ there appear to be no reports of these molecules forming inclusion complexes with drug ion‐pairs. The orientation of the ion‐pair in the triplex was thought to be significant as at least two amines of the polyamine tag were shown to protrude out of the complex, which was thought to be critical in enabling the polyamine to be transported by the PTS after the formation of the triplex.^[^ ^3^ ^]^ Direct measurements of the ion‐pair physical stability in the presence and absence of the CD were not performed because previous work by the authors had shown that without a fluorescent chromophore ion‐pair dissociation measurements were not possible using standard analytical techniques.^[^ ^6^ ^]^ However, the prolongation of the polyamine facilitated enhanced uptake of the drug, when it was delivered both in vitro and in vivo using the triplex, shown in subsequent sections, provided evidence that the triplex did indeed physically stabilize the ion‐pair in physiologically relevant fluids.

### 2.2 Theophylline Uptake in A549 Cells

To mimic the mixing of the commercial intravenous theophylline formulation with the blood before lung cell exposure, the test solutions, formulated to mimic the intravenous infusion conditions (pH 9.6), were mixed with HBSS (pH 7.4) in a 1:1 ratio v/v before application to the cells. This protocol was developed and explained more fully in previous work.^[^ ^6^ ^]^ The final pH of the test solutions after the mixing procedure was ≈8.3. At this pH, the theophylline alone, spermine alone and the theophylline‐spermine ion‐pair did not cause any reduction in cell viability at all the proposed cell accumulation assay test concentrations (see Figure S3, Supporting Information) after a 1 h incubation with the test solutions (note: the subsequent cell accumulation studies exposed the cells to the test compounds for 20 min).

Drug uptake studies were performed in A549 cells to determine the effect on theophylline uptake of spermine targeting of the PTS and ion pair stabilization by CD. Theophylline uptake alone was concentration and temperature‐dependent (see Figures S4 and S5, Supporting Information). At 4 °C the concentration of theophylline in the cells (nanomolar per μg of protein) was enhanced by 1.5‐fold and 2.5‐fold at 2 and 20 min, respectively, compared to equivalent experiments at 37 °C, which suggested that active efflux of theophylline limits intracellular concentration. Supporting this, the application of the P‐gp inhibitors elacridar and valspodar both doubled the theophylline concentration in the cells at 20 min (Figure S6, Supporting Information). This efflux process complicated the study of the triplex on theophylline uptake in the A549 cells because both the PTS and drug efflux transporters are energy‐dependent. There is a paucity of theophylline cell uptake data in the literature. No previous studies have suggested theophylline is subject to active transport, but molecular modeling studies described in detail in subsequent sections indicated that it is a P‐gp transporter substrate.^[^ ^22^ ^]^. Both elacridar and valspodar are listed by the FDA as specific inhibitors for the P‐gp transporter and the protocols used to apply them in this study have previously shown to be sufficient to reverse the P‐gp mediated efflux in vitro.^[^ ^23^, ^24^ ^]^ A contribution to theophylline efflux by other transporters cannot be excluded because of a lack of inhibitor specificity, e.g., BCRP is also inhibited by elacridar, and MRP2 is inhibited by valspodar, but it was considered unlikely as the expression of these transporters has not been reported in A549 cells.

**Figure 3:**
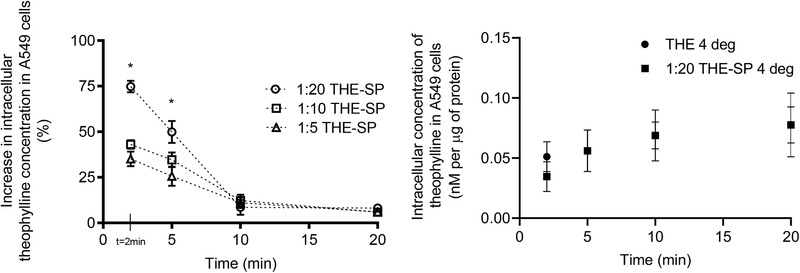
Left – The percentage increase in A549 cells intracellular theophylline at 37 °C when applied as a theophylline‐spermine (THE‐SP) ion pair at different molar ratios 1:20, 1:10, and 1:5. Right – A549 cell intracellular concentrations of theophylline (nanomolar per μg of protein) at 4 °C following the application of free theophylline (2.78 × 10^−6^ m) and theophylline spermine ion‐pair (2.78:55.6 × 10^−6^ m; 1:20 molar ratio) at 4 °C. Data represent *n* = 3 from three different cell flasks ±SD *statistically significant (*p* < 0.05) when compared to theophylline (Student's *t*‐test).

The theophylline spermine ion‐pair significantly (*p* < 0.05) increased intracellular theophylline compared to the drug alone (**Figure** **3**). This increase was greater when more spermine counter ion was present because the percentage of the theophylline complexed to the spermine increases until at a ratio of 1:20 at which point it is fully complexed (see Figure S1, Supporting Information). The pre‐treatment of the cells with spermine showed that there were no direct effects of the counter ion on the uptake of theophylline (see Figure S7, Supporting Information). There was a 74.8 ± 4.1% enhancement in theophylline concentration in the cells at 2 min for the 1:20 theophylline‐spermine molar ratio, 43.1 ± 2.4% for the 1:10 ratio and 35.1 ± 4.0% for the 1:5 ratio. However, in the absence of the CD, the enhanced theophylline accumulation in the cells was transient, lasting for less than 10 min because the ion‐pair dissociated reducing the uptake rate, and theophylline was removed from the cells by efflux. Although the PTS has been extensively studied, the gene(s) responsible for this transporter have not yet been identified and this made it impossible to confirm the transport of the ion‐pair via the PTS using molecular biology techniques. It has been suggested that there are multiple polyamine transport systems in mammalian cells with differing specificities that are capable of transporting polyamines and analogues.^[^ ^3^ ^]^ As polyamine decorated nanoparticles are actively taken up by the PTS one means by which the PTS may function is via endocytosis, but given that the PTS may take different forms it is also possible that the PTS on the A549 cells takes the form of a protein transporter with characteristics of a membrane “channel.”^[^ ^25^ ^]^

The activity of both P‐gp and PTS in the cell line experiments made it difficult to tease out the relative contributions of the two processes during the drug accumulation studies. However, lowering the experimental temperature to did discriminate between the two active processes. Reducing the temperature of the theophylline cell accumulation experiments from 37 to 4 °C negated the enhancement of theophylline uptake into the cells by the ion‐pair suggesting that the active uptake via PTS was more consequential to intracellular drug concentration than active efflux by the P‐gp as if it was the reverse reduction in temperature should have enhanced the ion‐pair effects (Figure 3). Ion‐pairing theophylline with spermine decreased the log P of the drug compared to the drug alone, thus it was unlikely that the intracellular concentrations of theophylline were increased by changes in partition into cell membranes (see Figure S8, Supporting Information).

**Figure 4:**
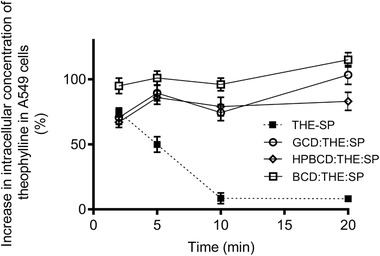
The percentage increase in A549 cell intracellular concentration of theophylline compared to free theophylline following the application of the theophylline‐spermine ion‐pair (THE‐SP) (2.78:55.6 × 10^−6^ m; 1:20 molar ratio) and THE‐SP ion‐pair in a triplex with gamma‐cyclodextrin (GCD:THE:SP, 2.78:2.78:55.6 × 10^−6^ m; 1:1:20 molar ratio), 2‐hydroxypropyl‐beta‐cyclodextrin (HPBCD:THE:SP, 2.78:2.78:55.6 × 10^−6^ m; 1:1:20 molar ratio) and beta‐cyclodextrin (BCD:THE:SP, 2.78:2.78:55.6 × 10^−6^ m; 1:1:20 molar ratio).

The CD theophylline‐spermine complexes maintained the elevated theophylline intracellular concentrations produced by the ion‐pair over the 20 min study period (**Figure** **4**). This suggested that the CD increased the physical stability of the ion‐pair when it was complexed with the CD and thus the triplex presented the PTS with a higher concentration of the drug associated with the targeting moiety over this time period. The sustained elevated intracellular drug concentrations were independent of CD type, which aligned with the NMR data that suggested all the CDs generated supramolecular triplexes with the theophylline‐spermine ion‐pairs. The pre‐treatment of the cells with CD showed that there was no direct effects of the CD on the uptake of theophylline thus its effects were assigned to an increase in the physical stability of the ion‐pair complex (see Figure S7, Supporting Information).

### 2.3 Molecular Modelling

**Figure 5:**
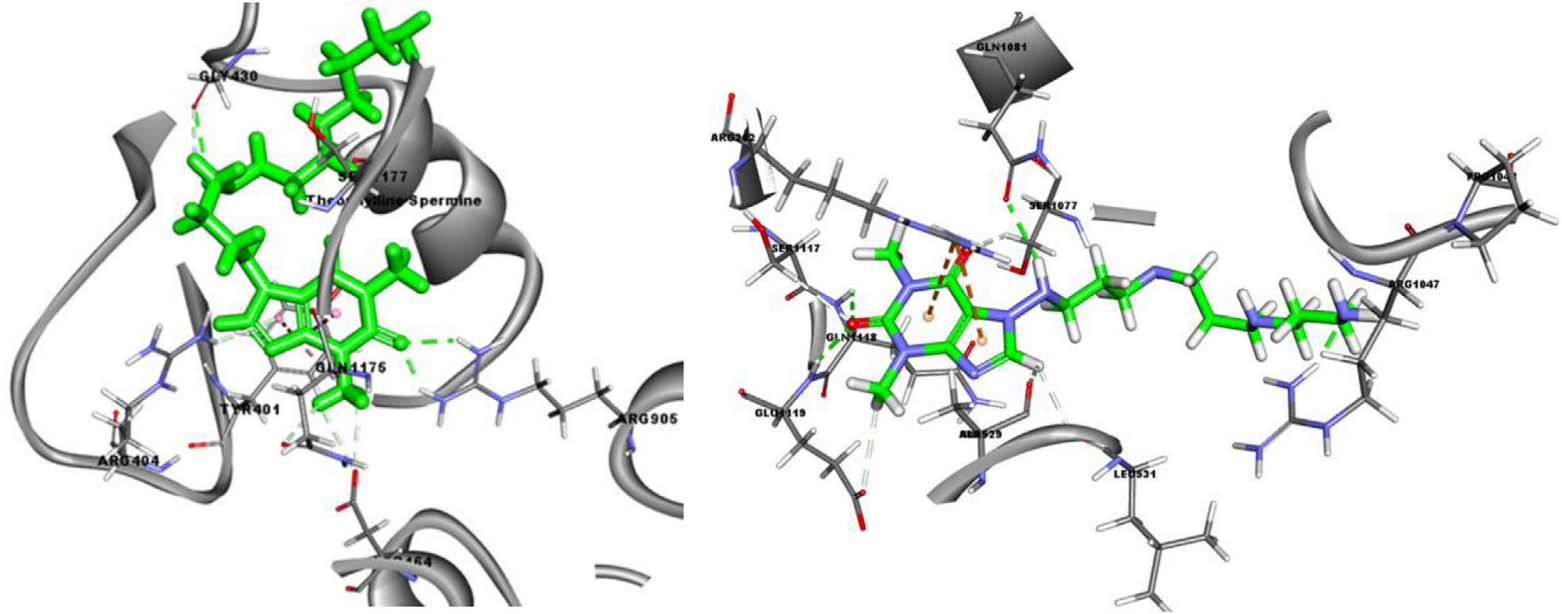
Interactions of Theophylline‐Spermine in P‐gp binding site 1 (left) and binding site 2 (right).

A molecular docking study was performed to probe the molecular‐level interaction between P‐gp and theophylline alone and ion paired with polyamines. This could not be done with the PTS as the structure has yet to be characterized although both are present in the A549 cell line.^[^ ^26^ ^]^ Previously identified ligand binding sites were considered for this study (Table S3, Supporting Information).^[^ ^27^ ^]^ Although the free energy of binding values observed for theophylline were considerably lower than those observed for other P‐gp substrates, e.g., the bronchodilator salbutamol (Figures S9 and S10, Supporting Information), theophylline showed sufficient molecular level interaction with both substrate binding sites of P‐gp to suggest it is a substrate.^[^ ^28^ ^]^ Ion‐pairing of theophylline with polyamines increased the binding affinity toward P‐gp compared to theophylline alone with a trend of increasing binding affinity as the length of the methylene linkers of the polyamines increased (Table S3, Supporting Information). The theophylline‐spermine ion‐pair showed free energy of binding of −31.15 and −33.00 kcal mol^−1^ for binding sites 1 and 2, respectively, as a consequence of forming six hydrogen bonds with amino acids in binding site 1 (Asp164, Arg404, Gly430, Arg905, Gln1175, and Ser1177 and hydrophobic interactions with Tyr401, **Figure** **5** – left) and eight hydrogen bonds were formed in binding site 2 (Ala529, Leu531, Arg1047, Ser1077, Gln1081, Ser1117, Gln1118, and Glu1119, Figure 5 – right).

The free energy of binding and the level of interaction with P‐gp substrate binding sites observed for the theophylline‐spermine ion pair were similar to those observed for salbutamol (Table S3, Supporting Information) suggesting that if the theophylline ion‐pair did not dissociate in the cell then it would be susceptible to P‐gp efflux.

### 2.4 Theophylline Biodistribution in Mice and Rats

**Figure 6:**
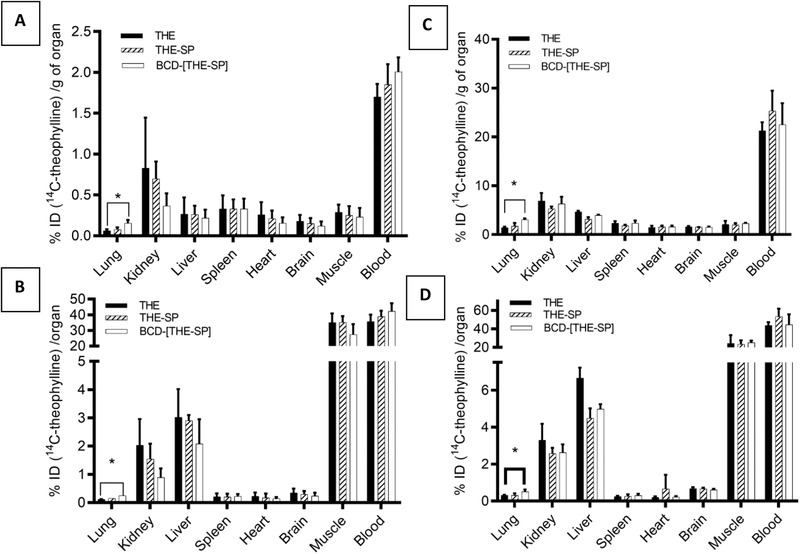
In vivo biodistribution profiles at *t* = 20 min following a slow intravenous injection of [^14^C]‐theophylline formulations in rats (left) and mice (right). Graphs A and C are presented as percentage of injected dose per g of organ (%ID/g organ) whilst graphs B and D are presented as percentage of injected dose per organ (%ID/organ) THE: theophylline alone, THE‐SP: theophylline‐spermine ion‐pair (1:20 molar ratio) and BCD‐THE‐SP: beta‐cyclodextrin‐theophylline‐spermine complex (1:1:20 molar ratio). *significantly different when compared to control group (*p* < 0.05) (Student's *t*‐test) (*n* = 3 ± SD).

The distribution of theophylline in vivo at *t* = 20 min calculated per whole organ following intravenous administration to mice showed a high proportion of drug in the blood (43.8 ± 3.5% for theophylline, 53 ± 4.2% for theophylline + spermine, 44.5 ± 11.2% for the triplex), by muscle (23.4 ± 3.5% for theophylline, 25.1 ± 2.2% for theophylline + spermine and 25.1 ± 2.2% for the triplex), liver (6.7 ± 0.6% for theophylline, 4.5 ± 0.5% for theophylline + spermine and 5.0 ± 2.6% for the triplex) and kidney (3.3 ± 0.9% for theophylline, 2.6 ± 0.3% for theophylline + spermine and 2.6 ± 0.4% for the triplex, **Figure** **6**). The distribution of theophylline in rats was very similar to that observed in the mouse. Previously it has been shown that theophylline readily distributes into muscle, which leads to a significant proportion of the drug residing in muscle due to its large volume.^[^ ^16^ ^]^ If biodistribution is normalized per g of organ, blood still has the highest proportion of administered dose, but the liver and kidney contain a higher concentration of drug compared to the muscle (Figure 6).

The lungs were the only organ in which drug biodistribution was altered by using the triplex formulation with significantly higher drug levels (*p* < 0.05) in both the lungs of mice and rats compared to when theophylline was administered alone. The increases were 2.2‐fold and 2.4‐fold in terms of % theophylline dose per g lung tissue (theophylline 1.4 ± 0.2% vs triplex 3.1 ± 0.3% in the mouse; theophylline 0.06 ± 0.02% vs triplex 0.16 ± 0.06% in the rat) and 1.7‐fold and 2.0‐fold in terms of % theophylline dose in the lungs (theophylline 0.3 ± 0.03% vs triplex 0.5 ± 0.1% in the mouse; theophylline 0.01 ± 0.08% vs triplex 0.02 ± 0.001% in the rat) in the mouse and rat, respectively. Unlike the triplex formulation, the intravenous administration of the theophylline‐spermine ion‐pair without the CD failed to increase the lung levels of theophylline in both animal species compared to theophylline alone (Figure 6, *p* > 0.05). These results illustrated that the theophylline ion‐pair, which had previously shown remarkable physical stability both in in vitro and ex vivo models of delivery to the lung was not stable in vivo.^[^ ^6^ ^]^

The expression of the PTS in different mammalian tissues has not been quantified, but functional studies have demonstrated a that there is a high level of PTS expression in the lungs.^[^ ^3^ ^]^ PTS substrates such as paraquat are known to distribute and even concentrate over time in the lung tissue, but they are also found in the liver, kidney and spleen.^[^ ^29^ ^]^ Less is known about the uptake of PTS substrates in these organs compared to the lungs, because they have been the subject of fewer studies investigating PTS mediated transport.^[^ ^3^ ^]^ In the absence of any enhancement of drug uptake in the brain, which expresses high levels of P‐gp, it appeared that any theophylline P‐gp efflux effects were not as important as the PTS uptake when delivered as a triplex, an effect that agreed with the conclusions from the cell line work performed in this study.^[^ ^30^ ^]^ The similar enhancement of triplex uptake in the mouse and the rat was also consistent with a PTS‐mediated mechanism because the polyamine uptake kinetics is very similar in these species (Rat; Km‐70 × 10^−6^ m, Vmax‐300 nmol g^−1^ tissue per hour; Mouse Km‐68 × 10^−6^ m, Vmax‐556 nmol g^−1^ tissue per hour). The rat is considered a good model for PTS uptake in humans due to its very similar polyamine uptake kinetics (Humans – Km – 40 × 10^−6^ m, Vmax‐ 300 nmol g^−1^ tissue per hour). It is noted that infection and inflammation have the potential to alter the disposition of drugs through modulation of drug transporters.^[^ ^31^ ^]^ However, any change in PTS expression in inflamed lungs would be difficult to assess and therefore no attempt was made to study uptake of the triplex mechanistically in inflamed lungs. Observational studies to determine theophylline disposition could be designed, but would require careful selection of appropriate animal model(s) with consideration of the severity and nature of the inflammation induced and the relevance to human disease.

The study design could be criticized for not using lung endothelial cells or smooth muscle cells for the uptake studies. However, a rat endothelial cell‐line with an active polyamine transport system has not been well characterized and therefore the A549 human alveolar epithelial cell line was selected instead.^[^ ^32^, ^33^ ^]^ Although the A549 cell line does not model the capillary endothelium the effects of the triplex in vitro, a 1.8‐fold increase in cell uptake, corresponded well with the 2.2‐fold and 2.4‐fold increases observed in the theophylline concentration in the lungs of mice and rats respectively compared to theophylline alone.

## 3 Conclusion

The in silico, in vitro, and in vivo data presented in this work demonstrated that spermine tagged theophylline‐CD supramolecular assemblies designed for uptake by the PTS can direct theophylline delivery to the lungs. CD was an essential component of the assemblies for enhancement in vivo as it enhanced the ion‐pair half‐life. Spermine was an effective targeting moiety for active uptake in lung cells, and the use of ion‐pairing (labile bond) to couple the targeting ligand to the drug was a useful strategy since it allowed ready drug release at the site of action. In the case of theophylline, where the systemic dose is limited by a very narrow therapeutic window (10–20 mg L^−1^ in the blood), using selective targeting to achieve a rapid 2‐fold enhancement in lung delivery may be a life‐saving intervention in a clinical emergency.

## 4 Experimental Section

### Materials

Theophylline (anhydrous, >99%), spermine (>99%), 1‐octanol, deuterium oxide (D_2_O) (D atom >99%), sodium hydroxide (NaOH), hydrochloric acid (HCl), sodium chloride (NaCl), all reagents for cell culture (RPMI‐1640 cell culture medium, fetal bovine serum (FBS), L‐glutamine, penicillin‐streptomycin, trypsin‐EDTA 0.25%, phosphate‐buffered solution (PBS), Hank's Balanced Salt Solution (HBSS), trypan blue and Triton TMX‐100), valspodar (>98%) and elacridar (>98%) were purchased from Sigma‐Aldrich, UK. Human lung epithelial A549 cells were obtained from the American Type of Culture Collection (ATCC), USA. 8[^14^C]theophylline (0.1 mCi mL^−1^, >98%) was purchased from American Radiochemical, USA. Optiphase “Safe” scintillation cocktail was from Fischer Scientific International, UK. All reagents for HPLC analysis were HPLC grade.

### NMR Measurements

^1^H‐NMR spectra were acquired at 300 ± 0.1 K using an Advance 400 MHz spectrometer (Bruker, UK) with a broadband inverse probe equipped with *x, y*, and *z* gradients. The chemical shifts were referenced to the D_2_O signal at 4.700 ppm that was consistent in all spectra measured. For each ^1^H NMR experiment, 16 transients were collected in 65 536 points over a 4010 Hz spectral window using a 1 s relaxation delay. Before Fourier transformation, the free induction decays (FIDs) were zero‐filled with 63 536 points and apodized by multiplication with an exponential decay to 1 Hz line broadening.

### Theophylline‐Spermine Association Strength

The NMR conditional binding constant (pK_NMR_) of the theophylline‐spermine ion‐pair was assessed by titrating spermine (concentration range 0–100 × 10^−3^ m) against theophylline (5 × 10^−3^ m) in D_2_O. The final pH of all mixtures was adjusted to 9.6 using HCl to mimic the theophylline intravenous administration formulation pH. Changes in the proton signals of theophylline upon mixing with spermine were used to establish the theophylline‐spermine association curve. The percentage of theophylline bound versus −log[spermine]_free_ were plotted and fitted with a regression model (Prism7, GraphPad USA). The pK_NMR_ of theophylline‐amines complexes was taken to be the concentration at 50% binding. Results were presented as the mean of *n* = 3 ± SD. No correction for the 0.41 unit difference between pD (when deuterium was used in the association studies) and pH values (used in the cell culture and in vivo experiments) was made since it made no important differences in the ionization of the molecules reported in the study, thus the term pH was used throughout when reporting the results.

### Ion‐Pair Cyclodextrin Complex Stoichiometry

A continuous variation method was employed to determine the ion‐pair CD complex stoichiometry following the previous studies, using ^1^H‐NMR.^[^ ^34^, ^35^ ^]^ The total concentration of CD and the theophylline‐spermine ion‐pair in the solutions was kept constant at 5 × 10^−3^ m. The molar fraction (*r*) of [CD]/[CD mixture] varied in the range of 0.1–0.9. The final pH of all mixtures was kept at 9.6 ± 0.1. Any pH adjustment was made using HCl. Results were presented as the mean of *n* = 3 ± SD.

### Cell Culture and Biocompatibility

Human lung epithelial A549 cells were used below twenty passages. The cells were maintained in a 95% humidified/ 5% CO_2_ atmosphere at 37 °C. They were grown in 75 cm^2^ flasks and cultured using RPMI‐1640 cell culture medium supplemented with 10% FBS, 0.3 g L^−1^ L‐glutamine, 100 μg mL^−1^ penicillin/streptomycin. The medium was changed every 2/3 days. When the cells reached 90% confluency (checked visually using a light microscope), they were sub‐cultured at a 1:3 split ratio using 0.25%trypsin/0.1% EDTA.

The cell biocompatibility of the individual compounds (theophylline, spermine, β‐CD, HP‐β‐CD, γ‐CD) and the mixtures with and without CD (i.e., with CD: CD‐theophylline‐spermine, without CD: theophylline‐spermine) in water adjusted to 9.6 ± 0.1 using 0.01 m NaOH/HCl was assessed using the MTT (3‐(4,5‐Dimethylthiazol‐2‐yl)‐2,5‐diphenyl‐tetrazolium bromide) test.^[^ ^36^ ^]^ Briefly, the cells were seeded into a 96‐well plate at a density of 1.0 × 10^4^ cells cm^−2^, allowed to grow for 24 h at 37 °C in a 5% CO_2_ atmospheric air incubator and rinsed three times with pre‐warmed PBS. For each biocompatibility test, 100 μL of pre‐warmed HBSS was added to each well followed by 100 μL of the test solutions, and the cells were incubated for 1 h at final concentrations using 2.78 × 10^−6^ m free theophylline. Untreated cells, i.e., cells incubated with cell culture medium only, were used as controls. After treatment, the cell media was discarded, replaced with 5 mg mL^−1^ MTT in 100 μL of cell culture medium solution, and incubated for 4 h. The cells were then lysed with 100 μL of lysis solution (10% SDS in DMF:water 50:50, pH 4.7) and the formazan formation quantified using a plate reader (Spectramax 190) at 570 nm (reference wavelength 690 nm). Relative cell viability was determined by dividing the optical density (OD) of treated wells over the OD of controls. Cells treated with 1% Triton X were used as a positive control whilst cells exposed to cell culture medium were used as a negative control. Experiments were repeated in triplicates (*n* = 3 plates for each treated group) and the data were presented as mean ± SD.

### Theophylline Uptake by A549 Cells

A549 cells were seeded onto a 12‐well plate at a density of 2 × 10^5^ cells cm^−2^. The culture medium was changed every other day until a confluent monolayer was established (approximately by 4 days post‐seeding), as determined visually using a light microscope. To investigate theophylline uptake, the cell culture medium was removed and the cells were washed three times with pre‐warmed PBS. The cells were then submerged in 0.5 mL pre‐warmed HBSS for 30 min. The uptake studies of theophylline were initiated by the application of 0.5 mL test solutions, containing [^14^C]‐theophylline. At 2, 5, 10, and 20 min, the cell media was carefully aspirated and the cell layers were washed three times with ice‐cold PBS. The cell layers were lysed by adding 1 mL of 1% Triton X solution for 45 min at 37 °C. The activity associated with the cell layers was determined by scintillation counting following the addition of scintillation cocktail. The protein content of each monolayer was determined using the BCA assay reagent kit. The results were expressed as the total accumulation of theophylline per microgram of protein. The experiments were performed at 37 and 4 °C. Each condition was studied in triplicate using three different flasks of cells and the data were presented as mean ± SD. For the P‐gp inhibition studies, a similar accumulation protocol was employed except that cells pre‐incubated with the P‐gp inhibitors (5 × 10^−6^ m of elacridar and 4 × 10^−6^ m of valspodar) for 30 min prior exposure to test solutions. Both inhibitors were first dissolved in DMSO due to their low solubility in HBSS and further diluted to the desired concentrations using HBSS (the final vehicle composition contained <0.1% v/v of DMSO).

### Molecular Modeling

Theophylline P‐gp molecular docking was performed using Dock Ligands (CDOCKER) protocol from Discovery Studio version 4.0. CDOCKER was an implementation of a CHARMm based docking tool where each orientation was subjected to simulated annealing molecular dynamics. The ligand poses were sorted by CHARMm energy (CDOCKER energy) and the top‐scoring (most negative, thus favorable to binding) poses are retained, where a higher negative value indicated a more favorable binding. This score includes internal ligand strain energy and receptor‐ligand interaction energy and was used to sort the poses of each input ligand. The ligands’ library was prepared using Prepare Ligands protocol. The newly published pdb code used for P‐gp (6C0V) was loaded from the Protein Data Bank. It was optimized and prepared through a Prepare Protein protocol. The Prepare Protein protocol prepares proteins for input into other protocols, performing tasks such as inserting missing atoms in incomplete residues, modeling missing loop regions, deleting alternate conformations, removing un‐needed waters, standardizing atom names and protonating titratable residues using predicted pKs. Theophylline was docked together with the ion‐paired theophylline‐polyamines (theophylline‐ethyl amine, theophylline‐ethylene diamine, theophylline‐spermine, and theophylline‐spermidine). Salbutamol was used as a positive control for the molecular docking as a well‐established P‐gp substrate. Five top poses for each ligand docked into the binding site were selected. The average of those five poses energies was used as the scoring function (CDOCKER Energy). The binding interactions for the best pose with the least energy were highlighted.

The ion‐pair CD association complex structure was visualized using molecular modeling. The atomic coordinates for theophylline and spermine were taken from the PubChem database.^[^ ^37^ ^]^ For β‐CD, the atomic coordinates were accessed through the Crystallography Open Database and taken from the dataset provided by.^[^ ^38^ ^]^ Modeling of the ternary inclusion complex was carried out using HyperchemTM and the structure was optimized using Polak–Ribiere conjugate gradient minimization of the potential energy to an RMS gradient of 0.001 kcal mol^−1^ Å^−1^. Images were generated using Discovery Studio Visualizer (Accelrys Inc, CA, USA).

### Theophylline Biodistribution in Mice and Rats

Male, BALB/c mice (25–30 g) and Wistar rats (280–350 g) were caged in groups of 2–4 with free access to water and food. A temperature of 19–22 °C was maintained, with a relative humidity of 45–65%, and a 12 h light/dark cycle. Animals were acclimatized for 7 days before each experiment. All procedures followed the 1989 UK Home Office “Code of Practice for the Housing and Care of Animals Used in Scientific Procedures.” In total 9 rats and 9 mice were randomized into 3 groups of 3 animals each. The first group (control) was injected with [^14^C]‐theophylline only. The second and third groups were injected with [^14^C]‐theophylline ion‐paired with spermine at 1:20 drug‐spermine molar ratio and [^14^C]‐theophylline complexed with β‐CD and spermine at 1:1:20 molar ratio, respectively. All formulations were prepared in sterile saline pH adjusted to 9.6 ± 0.1. Animals were slowly injected (over ≈70 s for rats and ≈15 s for mice) via the tail vein with 630 μg Kg^−1^ (50 μCi Kg^−1^) or 200 μg Kg^−1^ (50 μCi Kg^−1^) for rats and mice, respectively.

Blood was withdrawn by tail vein puncture and collected to a heparinized tube. Approximately 3 min after the infusion was completed, animals were euthanatized by intraperitoneal (i.p) injection of pentobarbital sodium (200 mg mL^−1^) (1 μL g^−1^ of an animal). This process took around 20 min hence the biodistribution at *t* = 20 min was assessed. To wash out the organs before harvesting PBS was perfused through the animals via the left ventricle (100 rpm) for 10 min and the heart, lungs, muscle (skeletal), liver, kidney, brain, and spleen were collected.

### Liquid Scintillation Counting

The whole organs were collected and homogenized using a tissue homogenizer in phosphate‐buffered saline adjusting the fluid volume for each organ based on the addition of 0.2 mL of liquid per 100 mg of tissue before radioactivity counting. An aliquot of the blood or 200 μL of each tissue homogenate was transferred into a 20 mL scintillation vial. A 1.0 mL aliquot of tissue solubilizer (Soluene 350) was added to each vial and shaken overnight at 55 °C, 15 mL of acidified scintillation cocktail (to eliminate chemiluminescence) was added to the vials and they were kept in the dark for 24 h before counting. Colored samples (i.e., liver, spleen, kidney, and heart) were decolorized with 300 μL of 30% hydrogen peroxide and 300 μL isopropanol to stop foaming. Samples were subsequently shaken at 55 °C for at least 3 h to expel H_2_O_2_ content and then mixed with the acidified scintillation cocktail. [^14^C] radioactivity was quantified for each sample using a LS6500 multi‐purpose scintillation counter (Beckman Coulter, Brea, USA). Total radioactivity in the blood and muscle was calculated based on the total blood volume and muscle mass, which is equivalent to 7% (for blood), 43% (for muscle mass) of the bodyweight of rats and 8.5% (for blood), 45.5% (for muscle mass) of the bodyweight of mice.^[^ ^39^, ^40^ ^]^ Results were expressed as the percentage of the injected dose per organ (%ID/organ) and the percentage of the injected dose per g tissue (%ID/g of the organ) (*n* = 3). Blood volume rather than blood weight was used in all calculations.

### Statistical Analysis

Data were presented as mean ± standard deviation (SD). Statistical significance was performed using an unpaired one‐tailed Student's *t*‐test (SigmaPlot13). A *p*‐value of < 0.05 was considered to be statistically significant.

## Conflict of Interest

The authors declare no conflict of interest.

## Supporting information

Supporting Information
