# Supplementary material for: A Cyclodextrin‐Stabilized Spermine‐Tagged Drug Triplex that Targets Theophylline to the Lungs Selectively in Respiratory Emergency
Source: Adv Ther (Weinh). 2020 Sep 25;3(12):2000153. doi: 10.1002/adtp.202000153 (PMC7536984; doi:10.1002/adtp.202000153)
Supplement: Supplementary file 1 — Supporting Information [file ADTP-3-0-s001.pdf]

Supporting Information

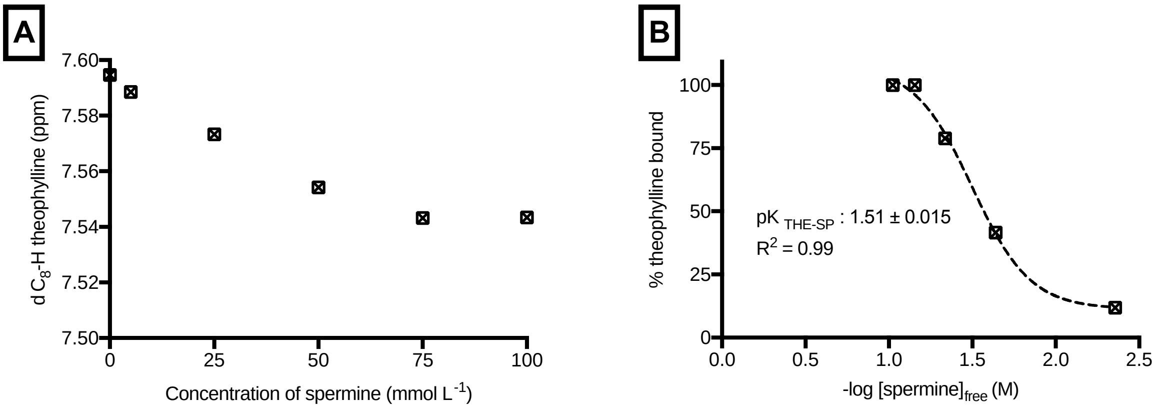

Figure S1. (A) The change of C_8-H of theophylline when mixed with increasing concentration of spermine in D_2O pH 9.6 ± 0.1 and (b) theophylline-spermine association curve. The values represent n=3 $\pm$ SD. Error bars are too small to be seen. The association curve was fitted with a Sigmoidal regression model (GraphPad Prism 7) to determine the THE-spermine NMR conditional binding constants (pK_{NMR}) which were determined at 50 % of bound theophylline.

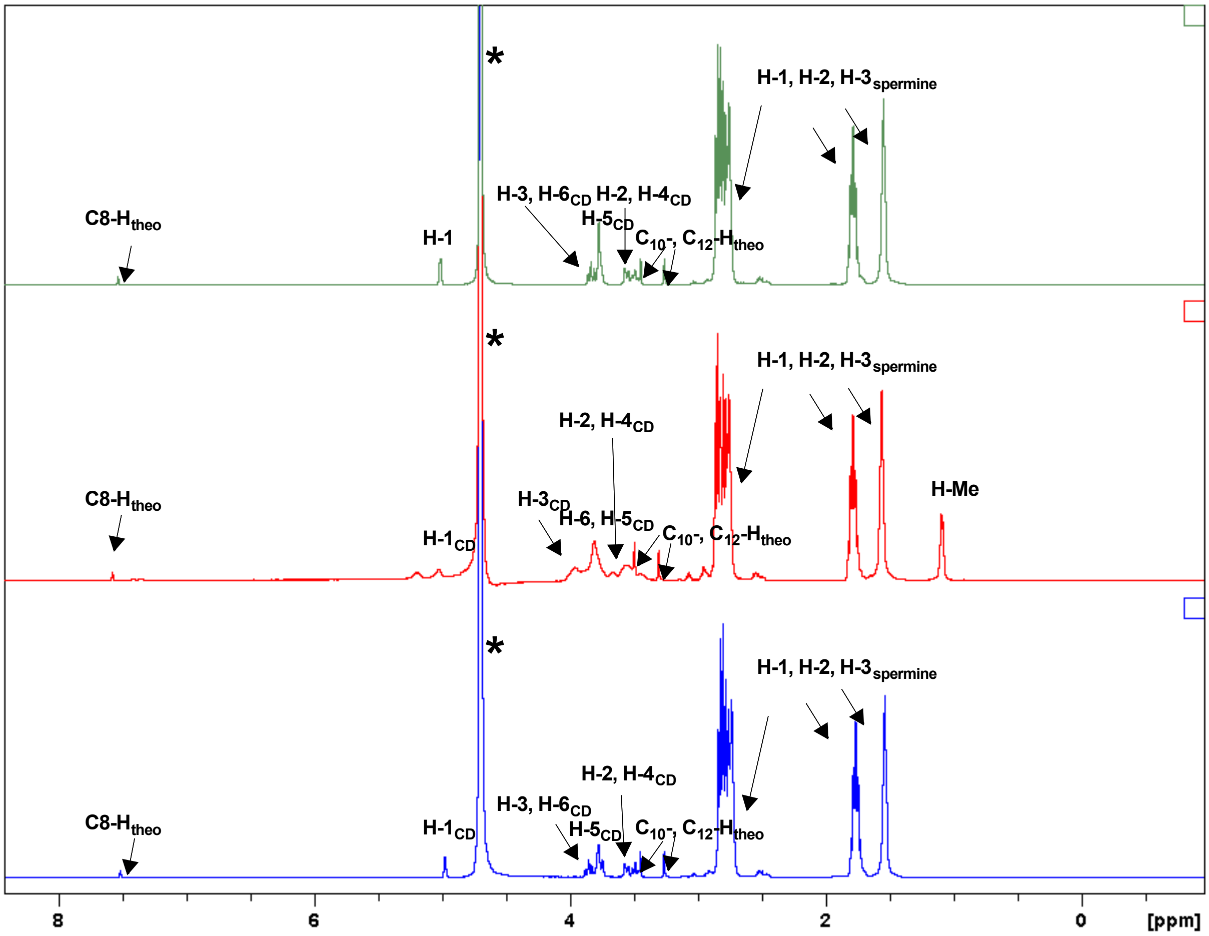

Figure S2. The ^{1}H-NMR spectra of β-CD-THE-SP (bottom graph), HP-β-CD-THE-SP (middle graph) and γ-CD-THE-SP (top graph) (CD-THE-SP molar ratio; 0.005:0.005:0.1 M) in D_2O pH 9.6 ± 0.1. * asterisk highlights the D_2O signal. CD-cyclodextrin, THE-theophylline, SP-spermine.

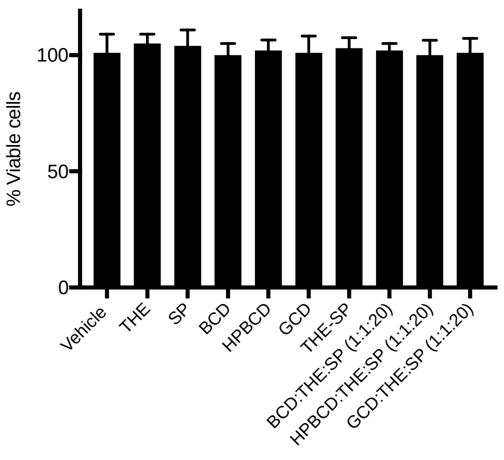

Figure S3. The percentage of viable A549 cells after 1h incubation at 37 °C with (i) 2.78 uM free theophylline, (ii) free spermine (55.6 μM), (iii) free cyclodextrins (2.78 μM), (iv) theophylline-spermine ion-pair (2.78:55.6 μM; 1:20 molar ratio) and (v) theophylline-spermine ion-pair complexed with cyclodextrins (2.78:2.78:55.6 μM; 1:1:20 molar ratio) to the HBSS-submerged cells assayed by MTT test. All solutions were prepared in water pH adjusted to 9.6. Values represent n=3 ± SD.

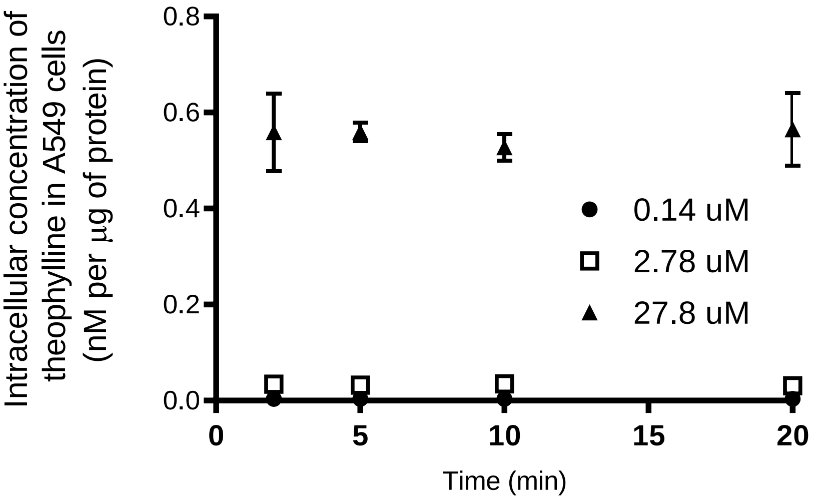

Figure S4. Total accumulation of theophylline in human lung epithelial A549 cells (nM per μg of protein) at 37°C the application of 0.14, 2.78 and 27.8 μM theophylline to the HBSS-submerged cells at 2, 5, 10 and 20 minutes. All solutions were prepared in water at pH 9.6 and mixed with HBSS on the cell surface (n=3 ± SD).

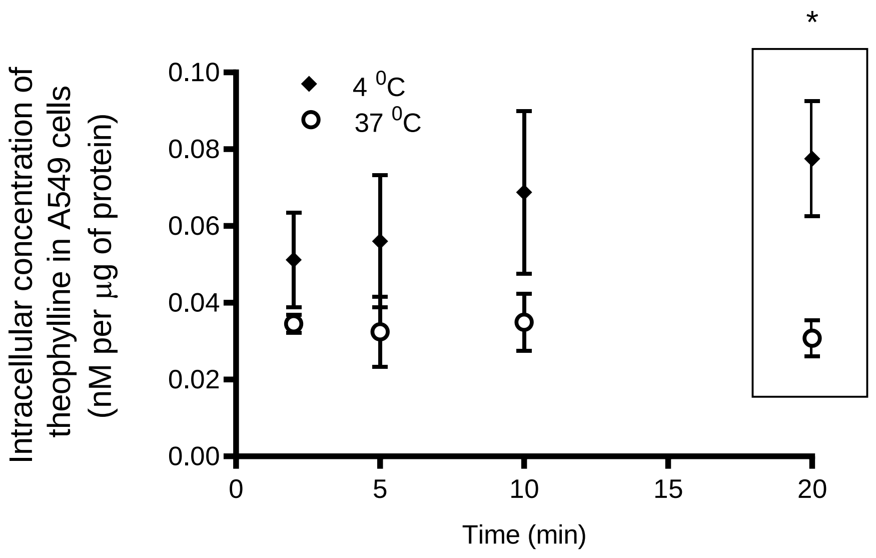

Figure S5. Total accumulation of theophylline in human lung epithelial A549 cells (nM per μg of protein) at 37 vs 4 °C following the application of 2.78 μM theophylline to the HBSS-submerged cells at 2, 5, 10 and 20 minutes. All solutions were prepared in water at pH 9.6 and mixed with HBSS on the cell surface (n=3 ± SD). *statistically significant ($p<0.05$) when compared the total accumulation of theophylline between 4 and 37 ^{0}C at t=20 min (Student's t-test).

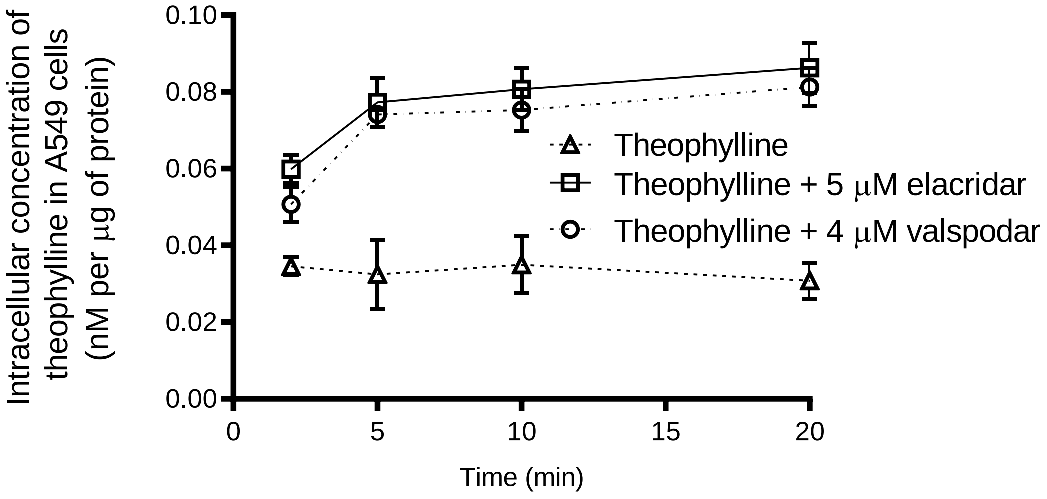

Figure S6. Effect of specific P-gp inhibitors (valspodar and elacridar) on the total accumulation of theophylline in A549 cells at 37 ^{o}C at 2, 5, 10 and 20 min. The figure shows the total accumulation of theophylline (nM per ug of protein) following the application of 2.78 uM of 0.5 mL theophylline prepared in water pH 9.6 to 0.5 mL HBSS-submerged cells after 30-min pre-incubation with the inhibitors. Cells treated with theophylline without pre-incubation with P-gp inhibitor was used as control (n =3±SD)

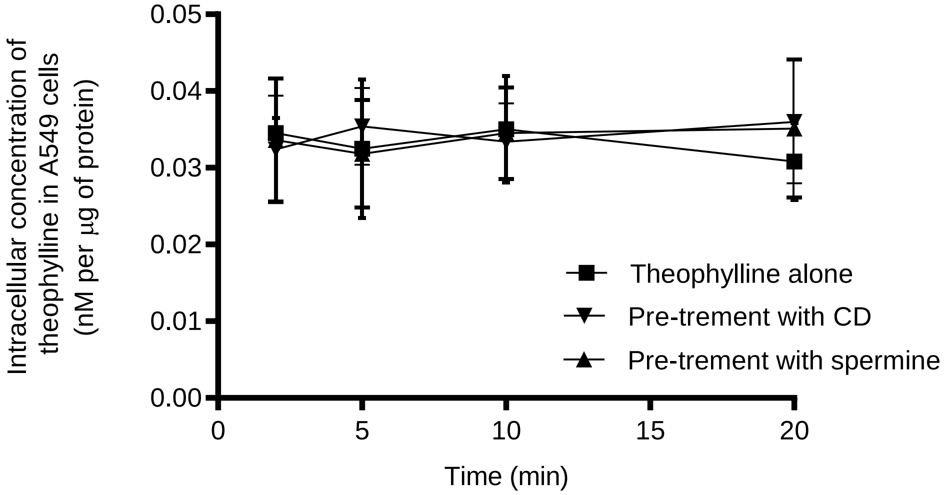

Figure S7. Total accumulation of theophylline (nmoles per µg of protein) in lung epithelial A549 cells at 37 °C following the application of free theophylline (2.78 µM) with and without spermine (27.8 µM) and CD (2.78 µM) pre-treatment (30 min) to HBSS-submerged cells at 2, 5, 10 and 20-min. All solutions were prepared in water pH adjusted to 9.6 (n=3 + SD).

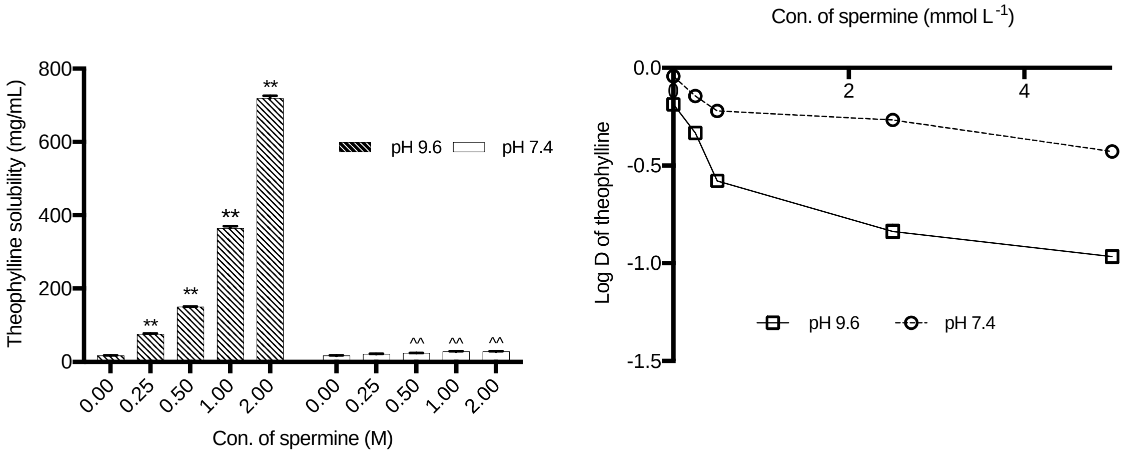

Figure S8. The aqueous solubility and log D profiles at pH 7.4 ± 0.2 and pH 9.6 ± 0.2 of theophylline in increasing concentrations of spermine. Both studies were performed at 37 ± 1 ^{o}C. Values represent means from n = 3 ± SD. Statistically significant^^/**($p < 0.001$) (One-way ANOVA) when compared to the solubility of free theophylline

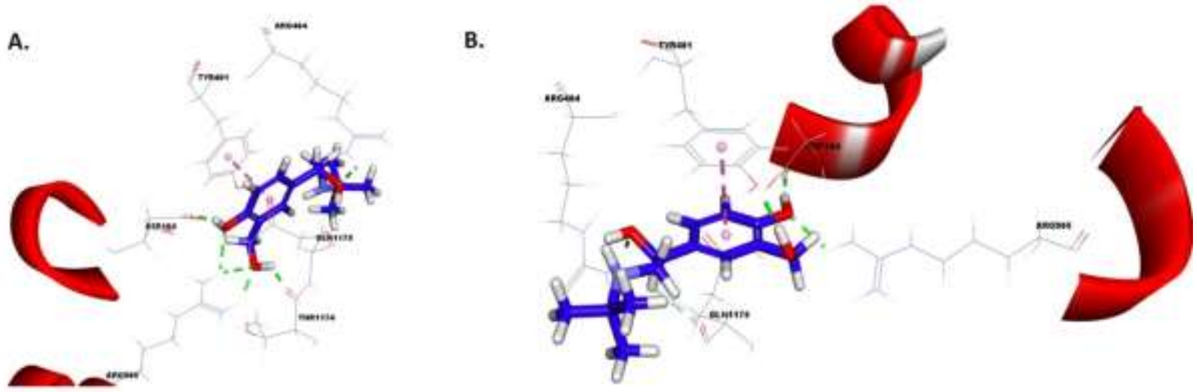

Figure S9. Interaction of (R)-(-)Salbutamol (A) and S)-(+)Salbutamol with the substrate binding site 1 of P-gp

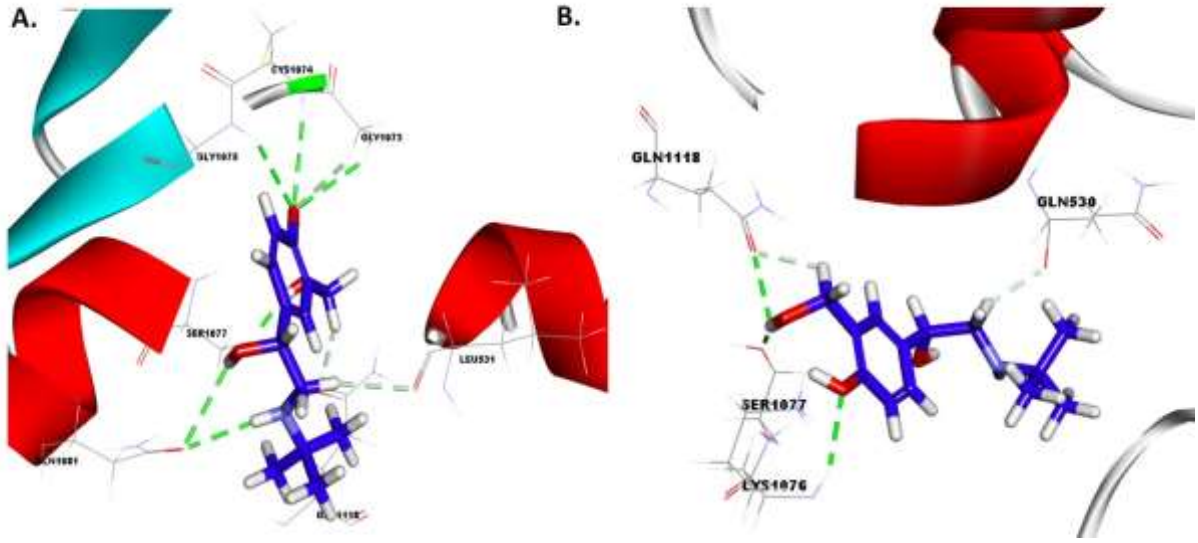

Figure S10. Interaction of (*R*)-(-)Salbutamol (A) and *S*)-(+)Salbutamol with the substrate binding site 2 of P-gp

Table S1. Chemical shifts of free theophylline (5 mM), spermine (100 mM) and theophylline-spermine mixture (5:100 mM)

Compound	C-H protons	Chemical shift (ppm)		
		Free	Complex (1:20)	Complex-Free
Theophylline	C_8-H	7.589	7.543	- 0.046
	C_{10}-H	3.303	3.297	- 0.006
	C_{12}-H	3.489	3.483	- 0.006
Spermine	H-1	1.821	1.822	- 0.001
	H-2	1.569	1.563	- 0.003
	H-3	2.882	2.879	- 0.004

Table S2. The chemical shifts, ∂ (ppm) of the free cyclodextrins and when formed the complex *i.e.,* cyclodextrin-theophylline-spermine complex (1:1:20 molar ratio) in D_2O pH 9.6 ± 0.1.

CD	C-H protons	Chemical shift (ppm)		
		Free CD	Complex	Complex-Free CD
HP- β-CD	H-1	5.198	5.177	- 0.021
	H-2	3.666	3.646	- 0.020
	H-3	3.971	3.949	- 0.022
	H-4	3.576	3.557	- 0.019
	H-5	3.816	3.795	- 0.021
	H-6	3.816	3.799	- 0.017
	H-Me	1.103	1.084	- 0.019
β -CD	H-1	5.014	4.978	- 0.036
	H-2	3.612	3.577	- 0.035
	H-3	3.933	3.881	- 0.052
	H-4	3.553	3.515	- 0.038
	H-5	3.824	3.778	- 0.046
	H-6	3.857	3.817	- 0.030
γ -CD	H-1	5.056	5.015	- 0.041
	H-2	3.619	3.577	- 0.042
	H-3	3.906	3.862	- 0.044
	H-4	3.560	3.519	- 0.041
	H-5	3.820	3.777	- 0.043
	H-6	3.855	3.815	- 0.038

Table S3. A summary of P-gp binding site energies for salbutamol (control P-gp substrate) T theophylline and theophylline ion-pairs. Mean data for 5 docking poses ± 1 SD).

Ligand	Binding Site 1 kcal/mol	Binding Site 2 kcal/mol
**(*R*)-(-)Salbutamol**	-32.88	-32.96
**(*S*)-(+)Salbutamol**	-35.51	-32.74
**Theophylline**	-14.98	-13.77
**Theophylline-Ethyl Amine**	-20.78	-17.45
**Theophylline-Ethylene Diamine**	-23.42	-21.27
**Theophylline-Spermidine**	-31.65	-32.27
**Theophylline-Spermine**	-31.15	-33.00
